# Universal quantum simulation of single-qubit nonunitary operators using duality quantum algorithm

**DOI:** 10.1038/s41598-021-83521-5

**Published:** 2021-02-17

**Authors:** Chao Zheng

**Affiliations:** Department of Physics, College of Science, North China University of Technology, Beijing, 100144 People’s Republic of China

**Keywords:** Quantum simulation, Quantum information, Quantum mechanics, Quantum physics, Qubits

## Abstract

Quantum information processing enhances human’s power to simulate nature in quantum level and solve complex problem efficiently. During the process, a series of operators is performed to evolve the system or undertake a computing task. In recent year, research interest in non-Hermitian quantum systems, dissipative-quantum systems and new quantum algorithms has greatly increased, which nonunitary operators take an important role in. In this work, we utilize the linear combination of unitaries technique for nonunitary dynamics on a single qubit to give explicit decompositions of the necessary unitaries, and simulate arbitrary time-dependent single-qubit nonunitary operator *F*(*t*) using duality quantum algorithm. We find that the successful probability is not only decided by *F*(*t*) and the initial state, but also is inversely proportional to the dimensions of the used ancillary Hilbert subspace. In a general case, the simulation can be achieved in both eight- and six-dimensional Hilbert spaces. In *phase matching* conditions, *F*(*t*) can be simulated by only two qubits. We illustrate our method by simulating typical non-Hermitian systems and single-qubit measurements. Our method can be extended to high-dimensional case, such as Abrams–Lloyd’s two-qubit gate. By discussing the practicability, we expect applications and experimental implementations in the near future.

## Introducition

Quantum operators^1^ take a basic role in quantum information processing as building blocks of quantum circuits and communication encoding. Series of quantum operations are able to undertake computing tasks^2–4^, evolve physical systems^1,5^ and simulate quantum phenomena^6,7^. A class of unitary quantum operators is investigated early on for reasons. One reason is that time-evolutions are naturally unitary in the conventional quantum mechanics. Another reason may due to the success of Grover’s square-root-accelerated searching algorithm^4^ which strengthens the viewpoint that the core of a algorithm is to design the step-by-step unitary evolution of the system^8^. Besides, it is sufficient to apply series of unitary operations to simulate a variety of Hermitian systems and related phenomena^5^, leading scientists to pay more attentions on the products of unitary operators.

However, unitary operators and their products are not enough to everything. On one hand, it is not easy to design new breakthrough quantum algorithms only by multiplying unitary operations. For example, it is believed that NP-complete problems cannot be solved in polynomial time by unitary quantum computing, while the nonunitary operation (such as Abrams–Lloyd’s gate) is possible^9,10^. On the other hand, the requirement of Hermiticity is unnecessary for some novel physical systems^11–16^, of which the time-evolution operators are no longer unitary. Besides, evolutions of open quantum systems or dissipative-quantum systems are nonunitary, which is important in modeling the system-environment interaction. Therefore, it is necessary to introduce nonunitary operators into quantum computing and simulation.

Quantum simulation provides an efficient and effective way to investigate nature by itself^17^. A Hermitian system can be simulated by the product of a series of unitary operators^1,5,18–20^, whereas it is neither suitable for non-Hermitian systems^21–30^ nor for dissipative-quantum systems^31–35^ of which the evolutions are non-unitary. Several efforts have been made to simulate dissipative-quantum systems. For examples, Barreiro et al. experimentally implement open-system dynamics through the dissipative map^32^; Hu et al. propose and demonstrate a general quantum algorithm to evolve open quantum by simulating Kraus maps^33^; Rost et al. simulate condensed matter system^34^; Viyuela et al. prepare topological thermal states to simulate a topological insulator open system for the topological-Uhlmann-phase measurement^35^. In recent years, non-Hermitian quantum mechanics and related systems are investigated massively. Typical ones among them are PT-symmetric, anti-PT-symmetric, pseudo-Hermitian, anti-pseudo-Hermitian systems, and etc. Motivations to investigate non-Hermitian systems include extending quantum theory, studying open quantum systems, discovering novel properties and applications. For example, Hermiticity has been seen to be a necessary condition for a long time to ensure quantum mechanics physically since it keeps the energy eigenvalues real. However, it is found to be a sufficient but not essential one for the reality of observables. The first attractive extension is PT-symmetric quantum mechanics^11–13^, and then pseudo-Hermiticity is pointed out to be a sufficient and necessary condition keeping the spectrum of a Hamiltonian purely real^14–16^. Subsequently, both the two classes of non-Hermitian systems^36–61^ and their anti-symmetric counterparts^62–72^ are investigated theoretically and experimentally to develop non-Hermitian theory and discover appealing features. Therefore, it is meaningful to investigate how to realize nonunitary operators in a controllable Hermitian system, so that non-Hermitian systems can be simulated on both small quantum devices and near-term quantum computers.

In this work, we utilize the linear combination of unitaries (LCU) technique^73–79^ for non-unitary dynamics on a single qubit, and simulate single-qubit nonunitary operators in subspaces of different-dimensional Hilbert spaces. The nonunitary operator *F*(*t*) can be a time-evolution operator $e^{-(i/\hbar )Ht}$ of a non-Hermitian two-state system, a single-qubit measurement, and etc. Unitary expansion (UE) of the operator is the key to our method, and we decide four *phase matching* conditions in which *F*(*t*) can be simulated by only two qubits. If none of the conditions is met, three qubits are necessary. Our method simulates nonunitary operators in an indeterministic way. We find that the successful probability is not only affected by *F*(*t*) and the initial state of the system, but also inversely proportional to the dimensions of the total used space. We apply our method to nonunitary time-evolution operators and an arbitrary single-qubit measurement that can be seen as a nonlinear operation. We also show how to extend our method to multi-qubit nonunitary operators by simulating the Abrams–Lloyd’s gate. Finally we analyze the complexities and discuss experimental implementations in NMR and quantum optical systems.

## Unitary expansions of a single-qubit nonunitary operator

In this section, we investigate unitary expansions of an arbitrary single-qubit nonunitary operator *F*(*t*). A unitary expansion with less terms saves qubit resource and increases the successful probability as we will show in the next section. Therefore, we aim at expanding *F*(*t*) by less unitary terms and give the criteria.

We write the most general form of a single-qubit nonunitary operator as

1 $$\begin{aligned} F(t)=\left[ \begin{array}{cc} f_{11}(t) &{} f_{12}(t)\\ f_{21}(t) &{} f_{22}(t) \end{array}\right] , \end{aligned}$$

of which each element can be a function of time *t*. *F*(*t*) can be the time-evolution operator $e^{-(i/\hbar )Ht}$ of a non-Hermitian system, a nonunitary operator applied to a non-Hermitian or open system, and etc. *F*(*t*) may any operator that shrinks, preserves or enlarges the norm of a quantum-state vector.

In general, *F*(*t*) can be expanded by four terms of Pauli matrices $\sigma _{0}$, $\sigma _{1}$, $\sigma _{2}$ and $\sigma _{3}$ as

2 $$\begin{aligned} F(t)=f_{0}\sigma _{0}+f_{1}\sigma _{1}+f_{2}(i\sigma _{2})+f_{3}\sigma _{3}, \end{aligned}$$

where the four UE-parameters are

3 $$\begin{aligned} f_{0}=f_{0}(t)=\frac{f_{11}+f_{22}}{2}, \quad f_{1}=f_{1}(t)=\frac{f_{12}+f_{21}}{2}, \quad f_{2}=f_{2}(t)=\frac{f_{12}-f_{21}}{2} \quad \text {and}\quad f_{3}=f_{3}(t)=\frac{f_{11}-f_{22}}{2}. \end{aligned}$$

Noticing that the $f_{k}$’s ($k=0,1,2,3$) are time-dependent complex functions, we rewrite them as

4 $$\begin{aligned} f_{k}=f_{k}(t)=\left| f_{k}\right| e^{i\theta _{k}} \end{aligned}$$

for convenience, where $\left| f_{k}\right|$ and $\theta _{k}$ are the norm and phase angle that may change with time *t*. The explicit forms of Pauli matrices are shown below

5 $$\begin{aligned} \sigma _{0}=\left[ \begin{array}{cc} 1 &{} 0\\ 0 &{} 1 \end{array}\right] ,\quad \sigma _{1}=\left[ \begin{array}{cc} 0 &{} 1\\ 1 &{} 0 \end{array}\right] ,\quad \sigma _{2}=\left[ \begin{array}{cc} 0 &{} -i\\ i &{} 0 \end{array}\right] ,\quad \sigma _{3}=\left[ \begin{array}{cc} 1 &{} 0\\ 0 &{} -1 \end{array}\right] . \end{aligned}$$

### Three UE-terms

In a general case, we find that *F*(*t*) can be expressed by three UE-terms as

6 $$\begin{aligned} F(t)=e^{i\theta _{0}}\left| f_{0}\right| \sigma _{0}+e^{i\theta _{3}}\left| c_{1}\right| U_{1}+e^{i\theta _{3}}\left| c_{2}\right| U_{2}. \end{aligned}$$

The three UE-parameters are time-dependent and can be expressed as complex functions of $f_{k}$’s ($k=0,1,2,3$) in Eq. (3), which the details are presented in the Supplementary Information. $f_{k}$’s can be arbitrary complex functions with no limits on the norms.

### Two UE-terms

The number of UE-terms of *F*(*t*) can be reduced further when the phase angles $\theta _{k}$’s of the UE-parameters $f_{k}(t)$’s ($k=0,1,2,3$) satisfy some conditions, which we call them *phase matching* conditions.

We find four *phase matching* conditions, of which the details are presented in the Supplementary Information. Whichever condition is met, *F*(*t*) can be expressed by two UE-terms as

7 $$\begin{aligned} F(t)=a_{0}V_{0}+a_{1}V_{1}, \end{aligned}$$

where $a_{k}$’s and the elements of $V_{k}$ ($k=0$ and 1) are complex functions of time *t*; $V_{0}$ and $V_{1}$ are in SU(2). The explicit forms of $a_{0}$, $a_{1}$, $V_{0}$ and $V_{1}$, varying in different conditions, are give in the Supplementary Information.

## Duality quantum simulation

For the nonunitary operator *F*(*t*), it cannot be simulated only by one qubit in a two-dimensional Hilbert space as a unitary case. We simulate *F*(*t*) in a two-dimensional subspace of a larger Hilbert space using duality quantum algorithm^73^, which enables linear combinations of unitary operations.

Duality quantum algorithm was proposed in 2002^73^ for the first time and developed fast^74–77^. Because of the abilities to realize both the products and linear combinations of unitary operations, it has become one of the strongest tool in designing quantum algorithms^78^. For example, an algorithm based on linear combinations of unitary operations to simulate Hamiltonian dynamics in a closed quantum system^79^ takes advantages over that based on product formulas. Recently, scientists apply it to design a full quantum algorithm for quantum chemistry simulation^80^ .

Based on our unitary expansions in the “Unitary expansions of a single-qubit nonunitary operator” and duality quantum algorithm, we investigate quantum simulation of *F*(*t*) in a general case and in *phase matching* conditions using different-dimensional Hilbert spaces. The hybrid-system protocols show principle of our method clearly, while pure-qubit protocols enable experimental implementations on both small quantum devices and near-term quantum computers.

### Eight-dimensional protocol

Recall Eq. (2), *F*(*t*) can be simulated in an eight-dimensional Hilbert space by both a qubit-qudit hybrid system and three qubits. Although this is not the most efficient protocol, it is clear to show the principle and how the unitary expansion of *F*(*t*) is linked to the protocol.

**Figure 1:**
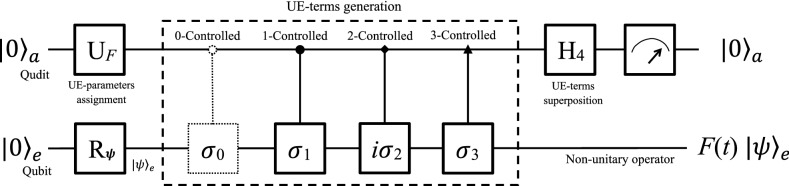
Quantum circuit to simulate *F*(*t*) by a qubit-qudit hybrid system. The system is initialized to $|0\rangle _{a}|0\rangle _{e}$, and the circuit is read from left to right. The qubit *e* can be prepared in an arbitrary state $|\psi \rangle _{e}$ by $R_{\psi }$. First, a single qudit rotation $U_{F}$ assigns the UE-parameters, and then four controlled operations generate the four UE-terms. Notice that the first $C_{0-\sigma _{0}}$ is unnecessary in practice but only for showing our theory clearly. Next, a Hadamard in SU(4) superposes the four UE-terms. Finally, a measurement is performed on the ancillary qudit to obtain the output $|0\rangle _{a}$ probabilistically, in which case the nonunitary *F*(*t*) is successfully simulated.

#### Using a qubit–qudit hybrid system

The hybrid system consists of a work qubit *e* and an ancillary four-dimensional qudit *a*, constructing the eight-dimensional Hilbert space. A qudit is a basic building block of high-dimensional quantum computers^1,81^. A four-dimensional qudit has four orthogonal logical bases $|0\rangle$, $|1\rangle$, $|2\rangle$ and $|3\rangle$, which can be realized by a four-state quantum system such as an ultra-cold atom with four non-degenerate-energy levels, a nuclear spin with four split-levels, a spin-(3/2) particle, and etc. In some quantum algorithm, qudits take advantages over qubits. For example, it reaches a higher accuracy to solve the eigenvalue problem using quantum phase estimation algorithm by qudits^82^ than by qubits^1^ (on page 217–226).

The quantum circuit is shown in Fig. 1. At the begining, the whole system is initialized to a pure state $|0\rangle _{a}|0\rangle _{e}$, and then the work qubit is prepared in a state $|\psi \rangle _{e}$ as needed by a single qubit rotation $R_{\psi }\in$ SU(2). Now we construct the nonunitary operation *F*(*t*), operating the work qubit with the assistance of the ancillary qudit. First, a single-qudit operator $U_{F}=\left( u_{jk}\right) \in$ SO(4) (where $u_{jk}$ are the matrix element and $k,j=$ 1, 2, 3, 4) is applied to the ancillary qudit. The explicit form of $U_{F}$ is not unique as long as $U_{F}^{\dagger }U_{F}=I_{4}$ and the first column vector is

8 $$\begin{aligned} \frac{1}{f}\left( \begin{array}{cccc} f_{0}&f_{1}&f_{2}&f_{3}\end{array}\right) ^{T}, \quad \text {where}\quad f=\sum \limits _{k=0}^{3} \sqrt{\left| f_{k}\right| ^{2}} \end{aligned}$$

is a normalizing factor. The rest column vectors can be obtained by the Gram–Schmidt orthogonalization. The effect of $U_{F}$ is to assign the UE-parameters $f_{k}$’s ($k=0,1,2,3$) to the probabilistic amplitudes of the ancillary qudit, which is linked to the first column vector of $U_{F}$. Second, four controlled gates follow, i.e., 0-controlled $\sigma _{0}$, 1-controlled $\sigma _{1}$, 2-controlled $i\sigma _{2}$ and 3-controlled $\sigma _{3}$, which the qubit and the qudit are the target and control ones. In practice, the first controlled gate can be removed because the effect is the same as $I_{4}$. The total effect of the controlled operators is to generate the four UE-terms in Eq. (2) and entangle them with the qudit. Third, a Hadamard operator $H_{4}\in$ SU(4) is applied to the ancillary qudit, which superposes the four UE-terms. Now, the whole system evolves to a superposition state

9 $$\begin{aligned} \frac{1}{2f}\left[ |0\rangle _{a}F(t)|\psi \rangle _{e}+f\sum \limits _{k=1}^{3} |k\rangle _{a}|s_{k}\rangle _{e}\right] . \end{aligned}$$

The explicit forms of $|s_{k}\rangle _{e}$’s are not given since they will be discarded if $|k\rangle _{a}$ is output (*k* = 1, 2, 3).

At last, a measurement is performed on the ancillary qudit. If the result outputs $|0\rangle _{e}$, the work qubit will be operated by the nonunitary in Eq. (1) to $F(t)|\psi \rangle _{e}$ (without a normalizing factor). Therefore, we simulate *F*(*t*) in an indeterministic way with a successful probability of

10 $$\begin{aligned} \frac{1}{4f^{2}} {}_e \langle \psi |F(t)^{\dagger }F(t)|\psi \rangle _{e}. \end{aligned}$$

The normalizing factor *f* in Eq. (8) affects the successful probability in Eq. (10) but doesn’t affect the effect *F*(*t*).

If the ancillary qudit is observed in one of the other three states $|k\rangle _{a}$, the work qubit will not be operated to $F(t)|\psi \rangle _{e}$ but to $|s_{k}\rangle _{e}$ ($k=1,2,3$). We will discard the results and re-initialize the hybrid system to the state $|0\rangle _{a}|0\rangle _{e}$. The whole process above is started over until $|0\rangle _{a}$ is obtained.

**Figure 2:**
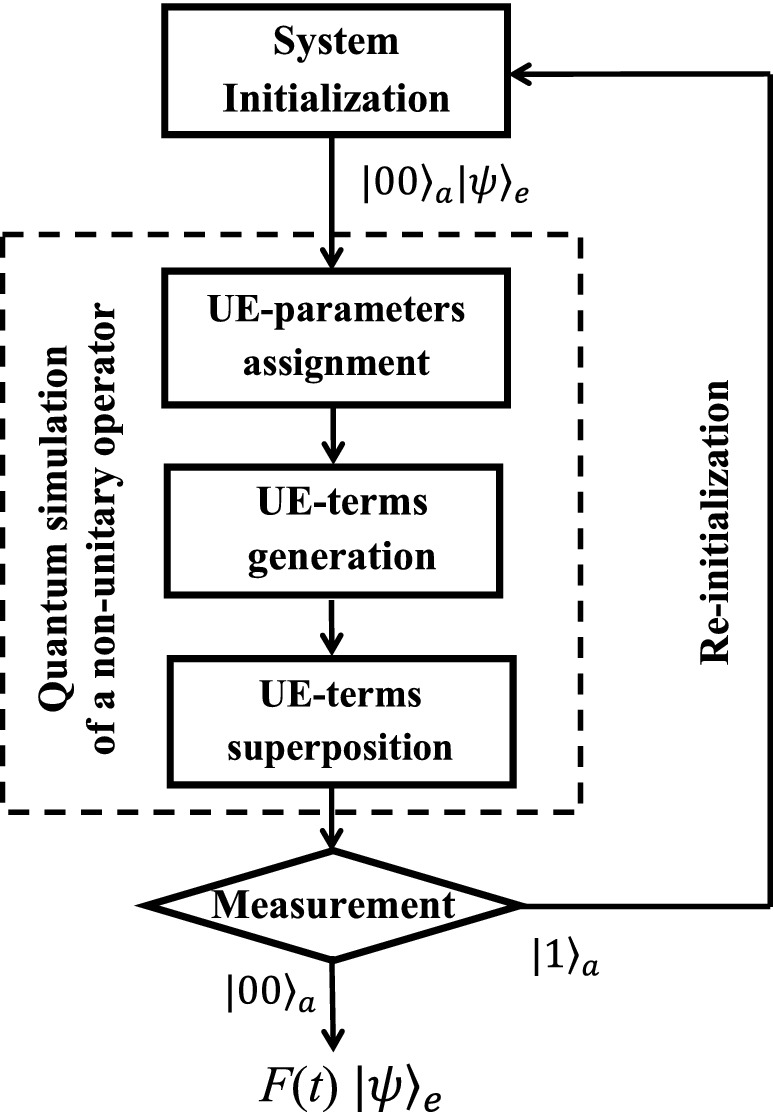
Flowchart to simulate *F*(*t*) in a quantum computer. The system is initialized in the first block. The second block includes UE-parameters assignment, UE-terms generation and superposition. Finally, measurements are performed on the ancillary subsystem to achieve the simulation of *F*(*t*) in an indeterministic way.

**Figure 3:**
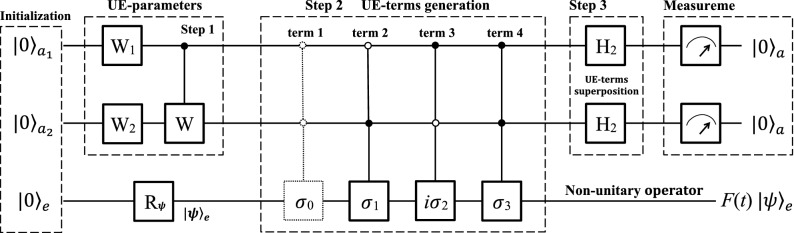
Quantum circuit for three qubits using the full Hilbert space. The three qubits is initialized to $|00\rangle _{a}|0\rangle _{e}$, and the qubit *e* can be rotated to an arbitrary state $|\psi \rangle _{e}$ by $R_{\psi }$ further. The first step is to assign the four UE-parameters, similar to $U_{F}$ in Fig. 1. Then four controlled-controlled operators are applied to generate the four UE-terms, where the first dashed one is unnecessary in practice. Next, two Hadamard gates are applied to the ancillary qubits to superpose the four UE-terms. Finally, measurements are performed on the ancillary qudits to simulate *F*(*t*) in an indeterministic way.

#### Using three qubits

To apply on a small quantum device or on a near-term quantum computer, we show how to simulate *F*(*t*) using two ancillary qubits and one work qubit. The flowchart of our quantum computer program is shown in Fig. 2. There are three main blocks, i.e., system initialization, nonunitary operation, and measurement.

The quantum circuit is shown in Fig. 3, reading from left to right. The system is initialized to a pure state $|00\rangle _{a}|0\rangle _{e}$, and a single-qubit rotation $R_{\psi }$ prepares the work qubit in an arbitrary state $|\psi \rangle _{e}$. The second block is the main one, including three steps of UE-parameters assignment, UE-terms generation and superposition.

In the first step, two single-qubit operators

11 $$\begin{aligned} W_{1}=\frac{1}{f}\left[ \begin{array}{cc} \sqrt{\left| f_{0}\right| ^{2}+\left| f_{1}\right| ^{2}} &{} -\sqrt{\left| f_{2}\right| ^{2}+\left| f_{3}\right| ^{2}}\\ \sqrt{\left| f_{2}\right| ^{2}+\left| f_{3}\right| ^{2}} &{} \sqrt{\left| f_{0}\right| ^{2}+\left| f_{1}\right| ^{2}} \end{array}\right] \quad \text {and}\quad W_{2}=\frac{1}{\sqrt{\left| f_{0}\right| ^{2}+\left| f_{1}\right| ^{2}}}\left( \begin{array}{cc} f_{0} &{} -f_{1}^{*}\\ f_{1} &{} f_{0}^{*} \end{array}\right) \end{aligned}$$

are applied to the first and second ancillary qubits, respectively. Then, a controlled gate

12 $$\begin{aligned} C_{1-W}=\left[ \begin{array}{cc} I_{2} &{} 0\\ 0 &{} W \end{array}\right] \end{aligned}$$

follows, where

13 $$\begin{aligned} W=W_{2}^{-1}\cdot W_{3} \quad \text {and}\quad W_{3}=\frac{1}{\sqrt{\left| f_{2}\right| ^{2}+\left| f_{3}\right| ^{2}}}\left[ \begin{array}{cc} f_{2} &{} -f_{3}^{*}\\ f_{3} &{} f_{2}^{*} \end{array}\right] . \end{aligned}$$

The above operations have a similar total effect as $U_{F}$ in Fig. 1, assigning UE-parameters $f_{k}$’s ($k=$1, 2, 3) to a superposition state of $|00\rangle _{a}$, $|01\rangle _{a}$, $|10\rangle _{a}$ and $|11\rangle _{a}$.

In the second step, four jointly-controlled gates are applied. The first dashed $C_{00-\sigma _{0}}$ gate in Fig. 3 is done naturally without any operation, while the last three controlled–controlled gates are

14 $$\begin{aligned} C_{01-\sigma _{1}}=\left[ \begin{array}{cccc} \sigma _{0} &{} 0 &{} 0 &{} 0\\ 0 &{} \sigma _{1} &{} 0 &{} 0\\ 0 &{} 0 &{} \sigma _{0} &{} 0\\ 0 &{} 0 &{} 0 &{} \sigma _{0} \end{array}\right] , \; C_{10-i\sigma _{2}}=\left[ \begin{array}{cccc} \sigma _{0} &{} 0 &{} 0 &{} 0\\ 0 &{} \sigma _{0} &{} 0 &{} 0\\ 0 &{} 0 &{} i\sigma _{2} &{} 0\\ 0 &{} 0 &{} 0 &{} \sigma _{0} \end{array}\right] \; \text {and} \quad C_{11-\sigma _{3}}=\left[ \begin{array}{cccc} \sigma _{0} &{} 0 &{} 0 &{} 0\\ 0 &{} \sigma _{0} &{} 0 &{} 0\\ 0 &{} 0 &{} \sigma _{0} &{} 0\\ 0 &{} 0 &{} 0 &{} \sigma _{3} \end{array}\right] , \end{aligned}$$

generating the four UE-terms and entangling them with the ancillary qubits.

In the third step, two Hadamard gates operate the two ancillary qubits, respectively. Now, the whole system evolves to a superposition state

15 $$\begin{aligned} \frac{1}{2f}\left[ |00\rangle _{a}F(t)|\psi \rangle _{e}+f \sum \limits _{kj=01}^{10,11} |k\rangle _{a}|s_{kj}\rangle _{e}\right] . \end{aligned}$$

The UE-terms are superposed in the first term as that in Eq. (2), while none of the rest three terms is linked to the initial state $|\psi \rangle _{e}$ by *F*(*t*).

Finally, measurements are performed on the ancillary qubits. If the two ancillary qubits are observed in a state $|00\rangle _{a}$, the work qubit will be operated by the nonunitary operation to a final state $F(t)|\psi \rangle _{e}$. If either of the two ancillary qubits is measured in $|1\rangle _{a}$, the work qubit will not be operated by *F*(*t*). The system will be re-initialized to $|00\rangle _{a}|0\rangle _{e}$ and the whole progress is started over until $|00\rangle _{a}$ is output. Similar to that using a hybrid system, it is in an indeterministic way to simulate *F*(*t*), and has the same successful probability as that in Eq. (10).

In fact, it is not necessary to expand *F*(*t*) by the Pauli operators in our protocol. If *F*(*t*) can be expanded by other UE-terms, our protocol is still applicable by replacing the Pauli operators with the relevant unitary operators in the quantum circuit above. On one hand, it is for convenience to illustrate by using the Pauli operators. On the other hand, it is more important to provide a method to reduce the number of UE-terms and thus increase the successful probability of our method, which is a key factor that whether the method has actual meaning to simulate multi-qubit nonunitary operators.

### Six-dimensional protocol

Now we show how to simulate *F*(*t*) in a six-dimensional Hilbert space based on our unitary expansion of *F*(*t*) in Eq. (6). It takes advantages over the previous protocols in two aspects. On one hand, it saves a two-dimensional subspace which may be used for other task at the same time. On the other hand, it has a higher successful probability than that using an eight-Hilbert space. Both qubit-qutrit hybrid and pure-qubit systems are able to achieve the simulation with the higher successful probability.

**Figure 4:**
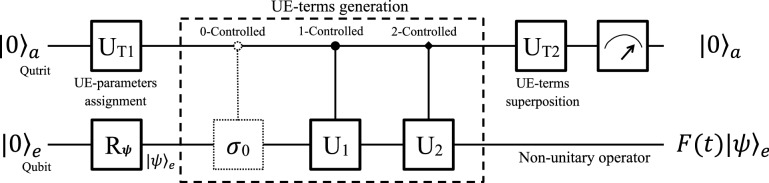
Quantum circuit for a qubit-qutrit hybrid system. The system is initialized to $|0\rangle _{a}|0\rangle _{e}$, and the qubit *e* can be prepared in an arbitrary state $|\psi \rangle _{e}$ by $R_{\psi }$. First, a single qutrit rotation $U_{T1}$ assigns the three UE-parameters, and then three controlled operators generate the three UE terms, where the first dashed $C_{0-\sigma _{0}}$ is not essential in practice. Next, another single-qutrit rotation superposes the three UE terms. Finally, a measurement is performed on the ancillary qudit to simulate the nonunitary *F*(*t*) probabilistically.

#### Using a qubit-qutrit hybrid system

We propose for the qubit–qutrit system to show the principle of this six-dimension protocol. A work qubit *e* and an ancillary qutrit *a* compose the hybrid system and constuct a six-dimensional Hilbert space. A qutrit is a three-state quantum system, e.g., three energy levels of an ultra-cold atom, a nuclear spin with three split-levels, a spin-1 particle, and etc. Any superposition states of it can be combined by the three orthogonal logical bases $|0\rangle$, $|1\rangle$ and $|2\rangle$. Similar to a qudit, a qutrit is also a building block of high-dimensional quantum computers.

The quantum circuit is shown in Fig. 4 for illustration, aiming at simulating $F(t)|\psi \rangle _{e}$ with the assistance of the ancillary qutrit. In the first block, the whole system is prepared in a pure state $|0\rangle _{a}|0\rangle _{e}$, and the work qubit is initialized to a state $|\psi \rangle _{e}$ by $R_{\psi }$ as needed. The second one is the main block to simulate *F*(*t*). First, a single-qutrit operator $U_{T1}\in$ SU(3),

16 $$\begin{aligned} U_{T1}=\frac{1}{f}\left[ \begin{array}{ccc} f_{0} &{} -\frac{fc_{2}^{*}}{\sqrt{\left| f_{0}\right| ^{2}+\left| c_{2}\right| ^{2}}} &{} \frac{f_{0}c_{1}^{*}}{\sqrt{\left| f_{0}\right| ^{2}+\left| c_{2}\right| ^{2}}}\\ c_{1} &{} 0 &{} -\sqrt{\left| f_{0}\right| ^{2}+\left| c_{2}\right| ^{2}}\\ c_{2} &{} \frac{ff_{0}^{*}}{\sqrt{\left| f_{0}\right| ^{2}+\left| c_{2}\right| ^{2}}} &{} \frac{c_{1}^{*}c_{2}}{\sqrt{\left| f_{0}\right| ^{2}+\left| c_{2}\right| ^{2}}} \end{array}\right] , \end{aligned}$$

is applied to the ancillary qutrit to assign the UE-parameters $f_{0}$, $c_{1}$ and $c_{2}$ in Eq. (6), where *f* is the renormalizing factor in Eq. (8). Second, three controlled operators generate the three UE-terms in Eq. (6). The first one is dashed because $C_{0-\sigma _{0}}$ is equal to the unit matrix $I_{3}$ in SU(3). Another two controlled operations,

17 $$\begin{aligned} C_{1-U_{1}}=\left[ \begin{array}{ccc} \sigma _{0} &{} 0 &{} 0\\ 0 &{} U_{1} &{} 0\\ 0 &{} 0 &{} \sigma _{0} \end{array}\right] \quad \text {and} \quad C_{2-U_{2}}=\left[ \begin{array}{ccc} \sigma _{0} &{} 0 &{} 0\\ 0 &{} \sigma _{0} &{} 0\\ 0 &{} 0 &{} U_{2} \end{array}\right] , \end{aligned}$$

act $U_{k}$ on the work qubit *e* when the ancillary qutrit is in state $|k\rangle _{a}$ ($k=1,2$), where $U_{1}$ and $U_{2}$ are that in Eq. (6).

At last, a single qutrit rotation

18 $$\begin{aligned} U_{T2}=\frac{1}{\sqrt{3}}\left[ \begin{array}{ccc} 1 &{} 1 &{} 1\\ \sqrt{\frac{3}{2}} &{} 0 &{} -\sqrt{\frac{3}{2}}\\ \frac{1}{\sqrt{2}} &{} -\sqrt{2} &{} \frac{1}{\sqrt{2}} \end{array}\right] \end{aligned}$$

is applied to the ancillary qutrit *a* to superpose the UE-terms. Now, the initial pure state $|0\rangle _{a}|\psi \rangle _{e}$ evolves to a superposition state

19 $$\begin{aligned} \frac{1}{\sqrt{3}f}\left[ |0\rangle _{a}F(t)|\psi \rangle _{e}+f \sum \limits _{k=1,2} |k\rangle _{a}|s'_{k}\rangle _{e}\right] . \end{aligned}$$

The first term is linked to *F*(*t*), while the rest terms are not.

We measure the qubit-qutrit hybrid system now. If the ancillary qutrit *a* is observed in a state $|0\rangle _{a}$, the work qubit *e* will evolve to $F(t)|\psi \rangle _{e}$ that entangled with $|0\rangle _{a}$. In this case, quantum simulation of *F*(*t*) is successful. Or, if the ancillary qutrit collapses into state $|1\rangle _{a}$ or $|2\rangle _{a}$, the results of work qubit $|s'_{1}\rangle _{e}$ and $|s'_{2}\rangle _{e}$ will be discarded. In these two cases, the system will be reset to the beginning, and then quantum simulation will be restarted until $|0\rangle _{a}$ is output. Therefore, it is also in an indeterministic way to simulate *F*(*t*), and the successful probability can be calculated as

20 $$\begin{aligned} \frac{1}{3f^{2}} {}_e \langle \psi |F(t)^{\dagger }F(t)|\psi \rangle _{e}, \end{aligned}$$

depending on both *F*(*t*) and $|\psi \rangle _{e}$. It is 4/3 times that of Eq. (10), meaning that the successful probability of this six-dimension protocol is higher than that of the eight-dimension one.

**Figure 5:**
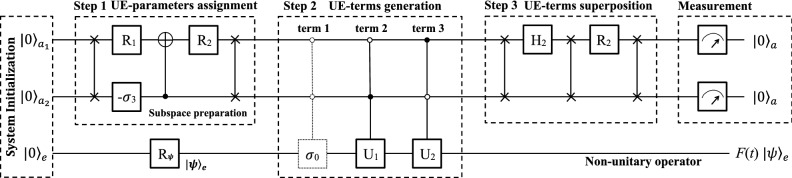
Quantum circuit for three qubits using a six-dimensional subspace. The three qubits is initialized to $|00\rangle _{a}|0\rangle _{e}$, and the qubit *e* is rotated to $|\psi \rangle _{e}$. The six-dimensional subspace is prepared and the UE-parameters are assigned in the first step, taking a similar role as the $U_{T1}$ in Fig. 4. Then the three UE-terms are generated by three controlled-controlled operators in the second step, where the first dashed one is unnecessary in practice. In the third step, a series of operations is applied to the ancillary qubits to superpose the three UE-terms as the effect of $U_{T2}$ in Fig. 4. Finally, measurements are performed on the ancillary qudits to simulate *F*(*t*) in a probabilistic way.

#### Using three qubits

The six-dimension protocol still involves three qubits to simulate $F(t)|\psi \rangle _{e}$. Instead of using the full Hilbert space, it uses a three-dimensional subspace of the two ancillary qubits. Figure 2 can be referred to as the flowchart of this protocol, which includes system initialization, simulation of nonunitary operation and measurement. Although the scheme is similar to that of the eight-dimension protocol, they are completely different in detail, which can be seen from the quantum circuit in Fig. 5.

At the beginning, the three qubits are initialized to a pure state $|00\rangle _{a}|0\rangle _{e}$, and $R_{\psi }$ rotates the work qubit to $|\psi \rangle _{e}$. The two ancillary qubits take a similar role as the ancillary qutrit of the hybrid system in the previous subsection. In the middle block, the nonunitary *F*(*t*) is simulated by three steps: space preparation and UE-parameters assignment, UE-terms generation, and UE-terms superposition. In the first step, a three-dimensional subspace of the ancillary qubits is prepared. Meanwhile, the three UE-parameters in Eq. (6) are assigned. In detail, the two ancillary qubits are swapped, and then two single-qubit operators $R_{1}$ and $-\sigma _{3}$ are applied to the first and second ancillary qubits, respectively. The explicit form of $R_{1}$ is

21 $$\begin{aligned} R_{1}=\frac{1}{f}\left[ \begin{array}{cc} c_{1} &{} -\sqrt{\left| f_{0}\right| ^{2}+\left| c_{2}\right| ^{2}}\\ \sqrt{\left| f_{0}\right| ^{2}+\left| c_{2}\right| ^{2}} &{} c_{1}^{*} \end{array}\right] , \end{aligned}$$

where $f_{0}$, $c_{1}$, $c_{2}$ and *f* are that in Eqs. (6) and (8). Next, a controlled-NOT gate is performed on the ancillary subsystem, which the first and the second qubits are the target and control ones respectively. After another single qubit rotation

22 $$\begin{aligned} R_{2}=\frac{1}{\sqrt{\left| f_{0}\right| ^{2}+\left| c_{2}\right| ^{2}}}\left[ \begin{array}{cc} c_{2}^{*} &{} f_{0}\\ -f_{0}^{*} &{} c_{2} \end{array}\right] \end{aligned}$$

is applied to the first ancillary qubit, the two ancillary qubits are swapped again. Now, the basis $|11\rangle _{a}$ of the ancillary subsystem is deleted, and the rest dimensions $|00\rangle _{a}$, $|01\rangle _{a}$ and $|10\rangle _{a}$ are left, constructing a six-dimensional subspace together with the work qubit. Meanwhile, the three UE-parameters in Eq. (6) are assigned to $|00\rangle _{a}|\psi \rangle _{e}$, $|01\rangle _{a}|\psi \rangle _{e}$ and $|10\rangle _{a}|\psi \rangle _{e}$, which is the key point in this step. The above operations have a similar effect as $U_{T1}$ performed on the ancillary qutrit in Fig. 4.

Based on the theory of three UE-terms, the second step aims at generating the three UE-terms $\sigma _{0}$, $U_{1}$ and $U_{2}$ in Eq. (6). Because $C_{00-\sigma _{0}}$ is a trivial unit matrix, only the two jointly-controlled gates $C_{01-U_{1}}$ and $C_{10-U_{2}}$ in Fig. 5 are necessarily in practice, of which the explicit forms are

23 $$\begin{aligned} C_{01-U_{1}}=\left[ \begin{array}{cccc} \sigma _{0} &{} 0 &{} 0 &{} 0\\ 0 &{} U_{1} &{} 0 &{} 0\\ 0 &{} 0 &{} \sigma _{0} &{} 0\\ 0 &{} 0 &{} 0 &{} \sigma _{0} \end{array}\right] \quad \text {and} \quad C_{10-U_{2}}=\left[ \begin{array}{cccc} \sigma _{0} &{} 0 &{} 0 &{} 0\\ 0 &{} \sigma _{0} &{} 0 &{} 0\\ 0 &{} 0 &{} U_{2} &{} 0\\ 0 &{} 0 &{} 0 &{} \sigma _{0} \end{array}\right] . \end{aligned}$$

Refer to Eq. (6) and the Supplementary Information for $U_{1}$ and $U_{2}$. Now, the three unitary terms are generated and entangled with the three bases of the ancillary subspace.

In the third step, the three UE-terms are superposed by swapping the two ancillary qubits three times with two single-qubit rotations $H_{2}$ and $R_{3}$ in between as shown in Fig. 5, where

24 $$\begin{aligned} R_{3}=\frac{1}{\sqrt{3}}\left[ \begin{array}{cc} \sqrt{2} &{} 1\\ 1 &{} -\sqrt{2} \end{array}\right] . \end{aligned}$$

Now, the whole system evolves to a superposition state

25 $$\begin{aligned} \frac{1}{\sqrt{3}f}\left[ |00\rangle _{a}F(t)|\psi \rangle _{e}+f \sum \limits _{k=01,10} |k\rangle _{a}|s'_{k}\rangle _{e}\right] , \end{aligned}$$

where the UE-terms are superposed in the first term as *F*(*t*). The rest terms in Eq. (25) are not shown explicitly because they are not linked to $F(t)|\psi \rangle _{e}$ and will be discarded after the measurements.

Finally, measurements are performed on the ancillary qubits. If the ancillary subsystem outputs a state $|00\rangle _{a}$, the work qubit will evolve to $F(t)|\psi \rangle _{e}$, meaning that the nonunitary operator is simulated successfully. If $|01\rangle _{a}$ or $|01\rangle _{a}$ is observed, the process will be terminated and started over again until $|00\rangle _{a}$ is observed. Therefore, it is an indeterministic protocol to simulate *F*(*t*). The successful probability is the same as that in Eq. (20). Therefore, not only a two-dimensional subspace are saved, but also the successful probability is higher than that in Eq. (10) using the full-eight dimensions.

We now analyze the complexities for the he 8D- and 6D-protocols by comparing the two quantum circuits in Figs. 3 and 5. In the circuit of the 6D-protocol, additional five two-qubit gates are introduced to prepare the subspace in step 1 and 3, but one controlled-controlled-$\sigma _3$, or $C^2(\sigma _3)$, is saved in step 2. According to Ref.^1^ (on page 182), five or more controlled-gates are necessary to implement the $C^2(\sigma _3)$. Therefore, the globe complexities of the two circuits to simulate a single-qubit nonunitary gate are nearly the same, though the local complexities in some steps are different.

**Figure 6:**
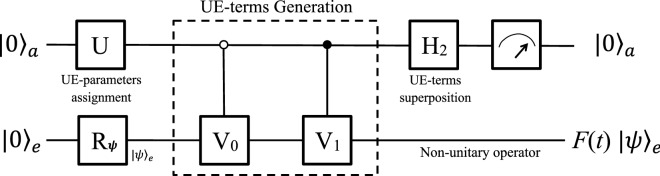
Quantum circuit using two qubits. The system consists of an ancillary and a work qubits, and the whole system is initialized to a state $|0\rangle _{a}|0\rangle _{e}$ . The work qubit can be further rotated to an arbitrary state $|\psi \rangle _{e}$ by $R_{\psi }$. First, a single-qubit rotation *U* is applied to the ancillary qubit to assign the two UE-parameters. Then, two controlled operations generate the two UE-terms. Third, a Hadamard superposes the UE-terms. Finally, the work qubit *e* will be operated by *F*(*t*), if the ancillary qubit is measured in $|0\rangle _{a}$.

### Four-dimensional protocol

Although three qubits are able to simulate single-qubit nonunitary operators universally, less qubits are preferred in some special cases for two reasons. One is to save qubit resource and decrease the complexity of quantum algorithm. The other reason is to increase the successful probability. Based on our unitary expansion theory of two UE-terms, *F*(*t*) can be expanded as Eq. (7) as long as one of the *phase matching* conditions is met, enabling us to simulate it using only two qubits.

Now, we show our four-dimensional protocol. The system consists of a work and an ancillary qubits, and the work qubit will be operated by *F*(*t*) with the assistance of the ancillary one. The quantum circuit is shown in Fig. 6, in which time proceeds from left to right.

At the beginning, the system is initialized to $|0\rangle _{a}|0\rangle _{e}$, and the work qubit is rotated to $|\psi \rangle _{e}$ by $R_{\psi }$ as needed. A single-qubit rotation

26 $$\begin{aligned} U=\frac{1}{f}\left[ \begin{array}{cc} a_{0} &{} -a_{1}^{*}\\ a_{1} &{} a_{0}^{*} \end{array}\right] , \end{aligned}$$

is applied to the ancillary qubit to assign the UE-parameters $a_{0}$ and $a_{1}$ in Eq. (7) to $|0\rangle _{a}|0\rangle _{e}$ and $|1\rangle _{a}|0\rangle _{e}$, respectively. The explicit forms of $a_{0}$ and $a_{1}$ are calculated for each* phase matching* condition (refer to the Supplementary Information), which depend on *F*(*t*) and always satisfy that

27 $$\begin{aligned} f=\sqrt{\left| a_{0}\right| ^{2}+\left| a_{1}\right| ^{2}}. \end{aligned}$$

Then two controlled gates,

28 $$\begin{aligned} C_{0-V_{0}}=\left[ \begin{array}{cc} V_{0} &{} 0\\ 0 &{} \sigma _{0} \end{array}\right] \quad \text {and} \quad C_{1-V_{1}}=\left[ \begin{array}{cc} \sigma _{0} &{} 0\\ 0 &{} V_{1} \end{array}\right] , \end{aligned}$$

are performed, where the explicit forms of $V_{0}$ and $V_{1}$ vary in * phase matching* conditions and have been given in the Supplementary Information. The two controlled gates generate the two UE-terms and entangle them with the ancillary qubit. Next, a Hadamard operation $H_{2}$ is applied to the ancillary qubit to superpose the two UE-terms, and the two-qubit system evolves to a state

29 $$\begin{aligned} \frac{1}{\sqrt{2}f}\left[ |0\rangle _{a}F(t)|\psi \rangle _{e}+|1\rangle _{a}\left( a_{0}V_{0}-a_{1}V_{1}\right) |\psi \rangle _{e}\right] . \end{aligned}$$

The first term of the entangled state is linked to *F*(*t*), while the second term will be discarded.

Finally, a measurement is performed on the ancillary qubit. If $|0\rangle _{a}$ is obtained, the work qubit *e* will be operated by the nonunitary *F*(*t*) with a successful probability of

30 $$\begin{aligned} \frac{1}{2f^{2}} {}_e \langle \psi |F(t)^{\dagger }F(t)|\psi \rangle _{e}, \end{aligned}$$

which depends on both the specific operator and the initial state that it is performed on. If the ancillary qubit is measured in state $|1\rangle _{a}$, the result of the work qubit will be discarded. If so, we start over the simulation until the output $|0\rangle _{a}$ is obtained. Although *F*(*t*) can also be simulated by the above protocols when it meets the *phase matching* conditions, the successful probability using two qubits is higher than that using three qubits. Therefore, it has benefits to judge whether the *phase matching* conditions are met before quantum simulation to save qubit and increase successful probability, so that the complexities and difficulties for experimental implementations can be decreased.

### Successful probability and complexity

#### Successful probability

From Eqs. (10), (20) and (30), we conclude that the successful probabilities to simulate a nonunitary *F*(*t*) are affected by three factors. The first one is *F*(*t*) itself, and the second one is the initial state $|\psi \rangle _{e}$ of the work qubit on which *F*(*t*) is performed. The third factor is the dimensions of the total used Hilbert space. The first two facts can be seen directly from ${}_e \langle \psi |F(t)^{\dagger }F(t)|\psi \rangle _{e}$ and *f*, while the third one is implicit.

In fact, we can rewrite Eqs. (10), (20) and (30) as a unified equation

31 $$\begin{aligned} \frac{dim_{e}}{dim}\cdot \frac{1}{f^{2}}\cdot {}_e \langle \psi |F(t)^{\dagger }F(t)|\psi \rangle _{e}, \end{aligned}$$

where *dim* is the dimensions of the total used Hilbert space including both the ancillary and work subsystems, and $dim_{e}=2$ is the dimensions of the work subspace on which *F*(*t*) is performed. The normalizing factor *f* is decided by the elements of *F*(*t*) in Eq. (8). Therefore, the successful probability is proportional to ${}_e \langle \psi |F(t)^{\dagger }F(t)|\psi \rangle _{e}$ and the dimensions of the work subspace, and meanwhile is inversely proportional to $f^{2}$ and the dimensions of the total used Hilbert space.

Equation (31) is also suitable for simulating a unitary operator by one qubit. In this case, both *dim* and $dim_{e}$ are equal to 2. Since *F*(*t*) are unitary in this case, the normalizing factor $f=1$ and $F(t)^{\dagger }F(t)$ becomes the unit in SU(2). Substituting the values into Eq. (31), the successful probability is equal to one. This means that a single-qubit unitary operator can be simulated by one qubit in a deterministic way, being accordance with the conventional quantum mechanics. Therefore, Eq. (31) is correct for both the unitary and nonunitary cases in which $dim_{e}=2$ and $dim=2,4,6$ or 8.

Our method can be generalized to simulate a high-dimensional or multi-qubit nonunitary operator and Eq. (31) is valid, which we will illustrate by the last example in the next section. From Eq. (31), the dimensions or number of qubits affect the successful probability by the first fraction ${dim_e}/{dim}$. Noticing the definitions of $dim_e$ and *dim*, this fraction is equal to the reciprocal of the used dimensions of ancillary subsystem, say $dim_a$, which depends on the numbers of UE-terms. Given that a *D*-dimensional nonunitary operator having $D^2$ matrix elements, a trivial unitary expansion needs about $O(D^2)$ UE-terms. In this case, the successful probability is tending to zero as $D\rightarrow 0$. However, if we can express the operator by *k* UE-terms (*k* is independent and much less than *D*), the first fraction in Eq. (31) is equal to a constant of $k^{-1}$. Therefore, one key point to increase the successful probability is to express the nonunitary operator by less UE-terms. We have investigated how to decrease UE-terms in details for single-qubit operators in the previous section, but it would be more complex for the case of high-dimensional or multi-qubit operators.

#### Complexity

To simulate *F*(*t*) using qubits, the complexity of a 2-dimensional protocol is less than that of the higher-dimensional protocols. The complexities of the 6- and 8-dimensional protocols are nearly the same in total but different in local steps. This may be led by the fact that, although the used dimensions are different, the two ancillary subsystems have the same dimension in total. Therefore, to simulate a single-qubit nonunitary operator using qubits, we can conclude that: (1) the complexity only depend on the number (but not the used dimensions) of the ancillary qubits; (2) The complexity will be increased as the number of the ancillary qubits increases. It is evident that the second conclusion is still hold to simulate higher-dimensional or multi-qubits nonunitary operators, while the first one should be investigated carefully in higher-dimension case.

Assuming that $q_a$ ancillary qubits are used to simulate a multi-qubits nonunitary operator *F*, we now analyze the complexity of the sub-space preparation by treating this process as an oracle, $B_{q_a}$ for $q_a$ ancillary qubits. One smaller oracle $B_{q_a -1}$ to assign parameters to $(q_a-1)$-ancillary qubits and $2^{q_a -1}$ $(q_a -1)$-qubit-controlled gates ($C^{q_a -1}(F)$) are needed to implement $B_{q_a}$. Because a $C^{(q_a -1)}(F)$ can be implemented by three $C^{(q_a -2)}(F)$ gates and two 2-qubit gate, a number of $O(12^{q_a -2})$ 2-qubit gates and *O*(1) single-qubit gates are necessary. Therefore, the complexity should be polynomial in the dimensions of the ancillary subsystem ($O[(2^{q_a})^l]$, where *l* is some positive number).

## Illustrations

We take time-evolution operators of several non-Hermitian two-state systems as examples to illustrate our unitary expansions and related protocols of quantum simulation. Quantum measurements can be seen as nonlinear operators in some way. Our method can be applied to simulate a single-qubit measurement in a four dimensional Hilbert space without annihilating the measured qubit. We apply our method to simulate Abrams–Lloyd’s gate, showing how it is extended to higher-dimensional cases.

### Non-Hermitian two-state systems

For some non-Hermitian Hamiltonians, a complex extension of the conventional quantum mechanics is developed by Bender et al.^13^. The time-evolution operator $e^{-(i/\hbar )Ht}$ keeps the unitarity as long as an appropriate inner product is constructed in the exact PT-symmetric phase.

We now simulate $e^{-(i/\hbar )Ht}$ for an arbitrary time-independent two-dimensional *H* by our method. Assuming the unitary expansion of *H* is

32 $$\begin{aligned} H=a_{0}\sigma _{0}+a_{1}\sigma _{1}+a_{2}\left( i\sigma _{2}\right) +a_{3}\sigma _{3}, \end{aligned}$$

where $a_{k}\in$ C ($k=0,1,2,3$). If we set $a=\sqrt{a_{1}^{2}+a_{2}^{2}+a_{3}^{2}}$, the operator $e^{-(i/\hbar )Ht}$ can be expanded as

33 $$\begin{aligned} e^{-\frac{ia_{0}}{\hbar }t}\left[ \cos \left( at/\hbar \right) \cdot \sigma _{0}-i\sin \left( at/\hbar \right) \sum \limits _{k=0}^{3} \frac{a_{k}}{a}\cdot \sigma _{k}\right] . \end{aligned}$$

Notice that *a* is a complex number in general, $\cos \left( at/\hbar \right)$ and $i\sin \left( at/\hbar \right)$ have definitions of $\left( e^{iat/\hbar }\pm e^{-iat/\hbar }\right) /2$. The four UE-parameters are

34 $$\begin{aligned} f_{0}(t)=e^{-ia_{0}t/\hbar }\cdot \cos \left( at/\hbar \right) \quad \text {and} \quad f_{k}(t)=-i\frac{a_{k}}{a}e^{-ia_{0}t/\hbar }\cdot \sin \left( at/\hbar \right) \quad \text {for} \quad k=1,2,3. \end{aligned}$$

To judge whether the *phase matching* conditions are met, the phase angles of $f_{0}(t)$ and $f_{k}(t)$’s ($k=1,2,3$),

35 $$\begin{aligned} \theta _{0}=\arg \left[ e^{-ia_{0}t/\hbar }\cdot \cos \left( at/\hbar \right) \right] \quad \text {and} \quad \theta _{k}=\arg \left[ -i\frac{a_{k}}{a}e^{-ia_{0}t/\hbar }\cdot \sin \left( at/\hbar \right) \right] , \end{aligned}$$

should be calculated based on $a_{k}$’s ($k=0,1,2,3$) in Eq. (32). If one of the *phase matching* conditions is met, the time-evolution operator can be simulated by two qubits. Or, three qubits should be involved to achieve the quantum simulation by the eight- or six-dimensional protocols, respectively.

To illustrate our theories, we investigate several typical non-Hermitian two-state systems with *PT*, anti-*PT*, *P*-pseudo and anti-*P*-pseudo Hermitian symmetries, of which the Hamiltonians are

36 $$\begin{aligned} H_{PT}=\left[ \begin{array}{cc} re^{i\theta } &{} se^{i\varphi }\\ se^{-i\varphi } &{} re^{-i\theta } \end{array}\right] , \; H_{APT}=i\left[ \begin{array}{cc} re^{i\theta } &{} se^{i\varphi }\\ se^{-i\varphi } &{} re^{-i\theta } \end{array}\right] , \; H_{PPH}=\left[ \begin{array}{cc} re^{i\theta } &{} s\\ u &{} re^{-i\theta } \end{array}\right] , \; \text {and} \quad H_{APPH}=i\left[ \begin{array}{cc} re^{i\theta } &{} s\\ u &{} re^{-i\theta } \end{array}\right] , \end{aligned}$$

satisfying $\left[ PT,H_{PT}\right] =0$, $\left\{ PT,H_{APT}\right\} =0$, $\left[ PT,H_{PPH}\right] =0$ and $\left\{ PT,H_{APPH}\right\} =0$, where $P=\sigma _{1}$ is the parity operator and *T* is the time-reversal operator having the effect that $i\rightarrow -i$.

The first three systems are found to meet the *phase matching* condition I, II, and both I and IV, and can be simulated by two qubits. For the last system, three qubits are necessary to achieve the simulation in a general phase, while two qubits are enough in some special cases. This conclusion is in accordance with a previous work^30^. Details of the UE-terms and parameters are presented in the Supplementary Information to demonstrate the statements above.

### Arbitrary single-qubit measurements

Quantum measurement can be seen as a quantum operation from the view of quantum information. Therefore, our unitary expansion method and simulation protocols can be applied to digitally simulate a single-qubit measurement in a four dimensional Hilbert space without annihilation of the work qubit.

It can be generalized to simulate an arbitrary single-qubit measurement *M*. Assuming the two orthogonal eigenstates of *M* are $|m_{\parallel }\rangle$ and $|m_{\perp }\rangle$, we can apply our unitary-expansion method to construct two nonunitary matrices

37 $$\begin{aligned} M_{\parallel }=|m_{\parallel }\rangle \langle m_{\parallel }|\quad \text {and}\quad M_{\perp }=|m_{\perp }\rangle \langle m_{\perp }|. \end{aligned}$$

The effect of the measurement *M* performed on $|\psi \rangle _{e}$ is equivalent to applying either $M_{\parallel }$ or $M_{\perp }$ to $|\psi \rangle _{e}$ with a probability of ${}_e \langle m_{\parallel }|\psi \rangle _{e}$ or ${}_e \langle m_{\perp }|\psi \rangle _{e}$, respectively. Notice that $M_{\parallel }\pm M_{\perp }$ are unitary, we perform a series of unitary operations of $U=H_{2}$, 0-controlled $\left( M_{\parallel }+M_{\perp }\right)$, 1-controlled $\left( M_{\parallel }-M_{\perp }\right)$ and another $H_{2}$ as quantum circuit in Fig. 6. The initial state $|0\rangle _{a}|\psi \rangle _{e}$ will evolve to a state

38 $$\begin{aligned} |0\rangle _{a}M_{\parallel }|\psi \rangle _{e}+|1\rangle _{a}M_{\perp }|\psi \rangle _{e}. \end{aligned}$$

We now measure the ancillary qubit. If an output $|k\rangle _{a}$ is obtained, the work qubit will evolve to $|k\rangle _{e}$ with a probability of ${}_e \langle k|\psi \rangle _{e}$ ($k=m_{\parallel },m_{\perp }$) and be kept for further use.

**Figure 7:**
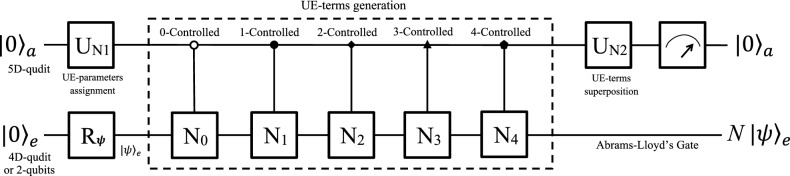
Quantum circuit to simulate Abrams–Lloyd’s gate. The system consists of a five-dimensional (5D) ancillary qudit and a four-dimensional (4D) work qudit (or two qubits), and is initialized to $|0\rangle _{a}|\psi \rangle _{e}$. UE-parameters are assigned by $U_{N1}\in$ SU(5), and then five controlled operations generate the related five UE-terms $N_{k}$ ($k=$0–4), which are superposed by $U_{N2}\in$ SU(5). Finally, a measurement is performed on the ancillary qudit. If it outputs $|0\rangle _{a}$, the Abrams–Lloyd’s gate *N* will be successfully applied to the work subsystem.

### Abrams–Lloyd’s gate

Our unitary-expansion method can be extended to simulate multi-qubit nonunitary operators. Here we take Abrams–Lloyd’s gate as an example for illustration.

Abrams and Lloyd investigated a nonlinear quantum algorithm^9^ to determine the existence of an input *x* such that $f(x)=1$. This algorithm is able to solve NP-complete problems using polynomial time in theory. The key technique of the nonlinear algorithm, which includes *n* input qubits and a flag qubit, is to iterate a nonlinear transformation to each of the *n* qubits and the flag qubit.

The nonlinear transformation has effects that $\frac{1}{\sqrt{2}}\left( |00\rangle +|11\rangle \right) \rightarrow \frac{1}{\sqrt{2}}\left( |01\rangle +|11\rangle \right)$, $\frac{1}{\sqrt{2}}\left( |01\rangle +|10\rangle \right) \rightarrow \frac{1}{\sqrt{2}}\left( |01\rangle +|11\rangle \right)$, and $\frac{1}{\sqrt{2}}\left( |00\rangle +|10\rangle \right) \rightarrow \frac{1}{\sqrt{2}}\left( |00\rangle +|10\rangle \right)$, which can be described by a two-qubit nonunitary gate (i.e., the Abrams–Lloyd’s gate)

39 $$\begin{aligned} N=\left[ \begin{array}{cccc} 0 &{} -1 &{} 1 &{} 0\\ 0 &{} 1 &{} 0 &{} 1\\ 0 &{} -1 &{} 1 &{} 0\\ 0 &{} 1 &{} 0 &{} 1 \end{array}\right] . \end{aligned}$$

We simulate this two-qubit nonunitary gate *N* as an example of higher-dimensional extension of our method. We find one unitary expansion of *N* is

40 $$\begin{aligned} N= \sum \limits _{k=0}^{4} n_{k}N_{k}, \end{aligned}$$

where the explicit forms of UE-terms $N_{k}$ and parameters $n_{k}$ ($k=0,1,2,3,4$) are given in the Supplementary Information. A system composed of two work qubits (or a four-dimensional work qudit) and a five-dimensional ancillary qudit is able to simulate the nonunitary gate *N*. The quantum circuit is shown in Fig. 7. First, the whole system is initialized to a pure state $|0\rangle _{a}|0\rangle _{e}$, and the work qubits are rotated to $|\psi \rangle _{e}$ as needed. The UE-parameters is assigned by a unitary $U_{N1}\in$ SU(5) of which the first column is

41 $$\begin{aligned} \frac{1}{f_{N}}\left( \begin{array}{ccccc} n_{0}&n_{1}&n_{2}&n_{3}&n_{4}\end{array}\right) ^{T}, \end{aligned}$$

where $f_{N}$ is a normalizing factor, calculated in the Supplementary Information. The rest column vectors can be obtained by the Gram–Schmidt orthogonalization. Then, five controlled gates $C_{k-N_{k}}$’s ($k=0,1,2,3,4$) are applied to generate the five UE-terms, which the ancillary qudit controls the work qubits. Next, another unitary operator $U_{N2}\in$ SU(5), of which the the first row is

42 $$\begin{aligned} \frac{1}{\sqrt{5}}\left( \begin{array}{ccccc} 1&1&1&1&1\end{array}\right) , \end{aligned}$$

is applied to the ancillary qudit to superpose the UE-terms. Now, the system evolves to a state

43 $$\begin{aligned} \frac{1}{\sqrt{5}f_{N}}\left[ |0\rangle _{a}N|\psi \rangle _{e}+f_{N} \sum \limits _{k=1}^{4} |k\rangle _{a}|s''{}_{k}\rangle _{e}\right] . \end{aligned}$$

Finally, a measurement is performed on the ancillary qudit. If it outputs $|0\rangle _{a}$, the Abrams–Lloyd’s gate is successfully applied to the two work qubits. If the output is not $|0\rangle _{a}$, the result will be discarded and the whole process will be started over until $|0\rangle _{a}$ is obtained.

The successful probability to simulate *N* is

44 $$\begin{aligned} \frac{1}{5f_{N}^{2}} {}_e \langle \psi |N^{\dagger }N|\psi \rangle _{e}. \end{aligned}$$

Given that the dimensions of the whole system and the two work qubits are twenty and four respectively, it is also in accordance with Eq. (31).

## Analyze for experimental implementations

From “Duality quantum simulation”, an arbitrary single-qubit nonunitary operator can be simulated by three qubits in general, and both the six- and eight-dimensional protocols can be adopted to realize the quantum simulation. Given that the stability and controllability of a real quantum system, the six-dimensional protocol is recommended because its successful probability is higher than that of the eight-dimensional one. When *phase matching* conditions are met, the four-dimensional protocol is preferred because it not only increase the successful probability but also save one qubit, decreasing difficulties for experimental implementations.

Both nuclear-magnetic-resonance (NMR) and quantum optics are candidates. For an NMR system, a nuclei of spin-(1/2) take the role as a qubit. The spatial-averaging method^83^ can initialize the system to a pseudo-pure state $|00\rangle _{a}|0\rangle _{e}$. Quantum gates are realized by sequences of magnetic pulses. Since arbitrary quantum gate can be divided into a series of single- and two-qubit gates^3^, we only need to analyze the two types of quantum gates. A single-qubit rotation is realized by a series of hard pulses, inducing small errors. Main errors are induced two-qubit operations, which are combined by hard pulses and free evolutions of two nuclei in periods of time^22^. Therefore, the four-dimensional protocol are recommended because it contains less two-qubit gates than other protocols.

Linear quantum optics system is another candidate for experimental implementation. Both two orthogonal polarized directions of a photon and two distinguished ways (positions) can take roles as qubits. We take the polarization qubit as an example. A single qubit operation can be realized by a series of half-wave and quarter-wave plates^84^. Although measurement induced nonlinearity^85,86^ can realize two-qubit gates in principle, the efficiency is extremely low especially when there are a lot of two-qubit gates. Therefore, an NMR system is recommended for experimental realizations of our protocols.

## Conclusions

We utilize the LCU technique for nonunitary dynamics on a single qubit, and simulate arbitrary time-dependent single-qubit nonunitary operator *F*(*t*) using duality quantum algorithm. We give explicit decompositions of the necessary unitaries, and minimize the number of unitary-expansion terms and the relevant operators for the single-qubit nonunitary evolutions. The successful probability not only depends on the specific parameters of *F*(*t*) and the initial state to which *F*(*t*) is applied, but also it is proportional to the ratio of the dimensions of the work qubit to the total used dimensions of the whole system. *F*(*t*) can be simulated in an eight-, six- or four-dimensional Hilbert space, depending on our unitary-expansion theory and *phase matching* conditions. In general, three qubits are enough to simulate arbitrary *F*(*t*) either using a six-dimensional subspace or the full space, and the successful probability of the former one is higher than that of the later one. In one of the four *phase matching* conditions, we find *F*(*t*) can be simulated by only two qubits with a higher successful probability. Therefore, it is necessary to judge whether the *phase matching* conditions are met before quantum simulation to improve the efficiency.

We illustrate with examples of typical non-Hermitian systems, such as (anti-)PT-symmetric, (anti-)*P*-pseudo-Hermitian and a general non-Hermitian Hamiltonians, expanding the time-evolution operators by unitary terms, checking the *phase matching* conditions, and choosing related protocols of quantum simulation. We also simulate a single-qubit measurement which can be seen as a nonunitary operation using our method. Our method can be extended to simulate a single-qudit or multi-qubit nonunitary operator, and we apply it to the Abrams–Lloyd’s gate as an example. Quantum simulation of single-qubit nonunitary operators is able to be implemented by our protocols at current stage. We will extend our method to simulate higher-dimensional nonunitary operator and optimize the UE-terms in the future.

## Supplementary information

Supplementary information.
