# Supplementary material for: Universal quantum simulation of single-qubit nonunitary operators using duality quantum algorithm
Source: Sci Rep. 2021 Feb 17;11:3960. doi: 10.1038/s41598-021-83521-5 (PMC7889913; doi:10.1038/s41598-021-83521-5)
Supplement: Supplementary file 1 — Supplementary information. [file 41598_2021_83521_MOESM1_ESM.pdf]

# Supplementary Information: Universal quantum simulation of single-qubit nonunitary operators using duality quantum algorithm


**Chao Zheng**

Department of Physics, College of Science, North China University of Technology, Beijing 100144, P. R. China.

czheng@ncut.edu.cn


### 2.1 Three UE-terms.

In a general case, we find that $F(t)$ can be expressed by three UE-terms as

$$F(t) = e^{i\theta_0}|f_0|\sigma_0 + e^{i\theta_3}|c_1|U_1 + e^{i\theta_3}|c_2|U_2. \tag{1}$$

The three UE-parameters can be expressed as functions of $f_k$'s in Eq. (**??**), which the details are presented in the Supplementary Information.

The three UE-parameters are $f_0$,

$$c_1 = e^{i\theta_3}\sqrt{|f_3|^2 + |f_1|^2\cos^2\varphi_1 + |f_2|^2\sin^2\varphi_2} \tag{2}$$

and

$$c_2 = e^{i\theta_3}\sqrt{|f_1|^2\sin^2\varphi_1 + |f_2|^2\cos^2\varphi_2}, \tag{3}$$

where

$$\varphi_1 = \theta_1 - \theta_3 \quad \text{and} \quad \varphi_2 = \theta_2 - \theta_3. \tag{4}$$

$U_1$ and $U_2$ are two unitary operators in SU(2),

$$U_1 = \begin{bmatrix} \cos\zeta_1 & e^{i\phi_1}\sin\zeta_1 \\ e^{-i\phi_1}\sin\zeta_1 & -\cos\zeta_1 \end{bmatrix} \quad \text{and} \quad U_2 = \begin{bmatrix} 0 & e^{i\phi_2} \\ -e^{-i\phi_2} & 0 \end{bmatrix}, \tag{5}$$

where $\phi_1$ is decided by

$$\cos\phi_1 = \frac{|f_1|\cos\varphi_1}{\sqrt{|f_1|^2\cos^2\varphi_1 + |f_2|^2\sin^2\varphi_2}} \quad \text{and} \quad \sin\phi_1 = \frac{|f_2|\sin\varphi_2}{\sqrt{|f_1|^2\cos^2\varphi_1 + |f_2|^2\sin^2\varphi_2}}; \tag{6}$$

$\phi_2$ is decided by

$$\cos\phi_2 = \frac{|f_2|\cos\varphi_2}{|c_2|} \quad \text{and} \quad \sin\phi_2 = \frac{|f_1|\sin\varphi_1}{|c_2|}; \tag{7}$$

$\zeta_1$ is decided by

$$\cos\zeta_1 = \frac{|f_3|}{|c_1|} \quad \text{and} \quad \sin\zeta_1 = \frac{\sqrt{|f_1|^2\cos^2\varphi_1 + |f_2|^2\sin^2\varphi_2}}{|c_1|}. \tag{8}$$

Notice that $f_k$ and $\theta_k$ $(k = 0, 1, 2, 3)$ are time-dependent, the variables above are implicit functions of time $t$.

## Four phase matching conditions for two UE-terms

As claimed in the main text, $F(t)$ can be expressed by two UE-terms as

$$F(t) = a_0 V_0 + a_1 V_1, \tag{S1}$$

as long as one of the four *phase matching* conditions are met. We now give details of the four *phase matching* conditions and the related parameters and matrices for Eq. () in the main text.

**Condition I.** If the two phase angles $\theta_0$ and $\theta_3$ in the maintext satisfy

$$(\theta_3 - \theta_0) \mod \pi = 0, \tag{S2}$$

the explicit forms of the UE-parameters are

$$a_0 = |a_0|\, e^{i\theta_0} = e^{i\theta_0}\sqrt{|f_0|^2 + |c_2|^2} \qquad \text{and} \qquad a_1 = c_1 \tag{S3}$$

referring $c_1$ and $c_2$ in the main text.

The two unitary matrices are

$$V_0 = \begin{bmatrix} \cos\zeta_0 & e^{i(\phi_2+\theta_3-\theta_0)}\sin\zeta_0 \\ -e^{-i(\phi_2+\theta_3-\theta_0)}\sin\zeta_0 & \cos\zeta_0 \end{bmatrix} \qquad \text{and} \qquad V_1 = U_1, \tag{S4}$$

where $\zeta_0$ is decided by

$$\cos\zeta_0 = \frac{|f_0|}{|c_0|} \quad \text{and} \quad \sin\zeta_0 = \frac{|c_2|}{|c_0|}; \tag{S5}$$

$U_1$ and $\phi_2$ are referred to that in the main text.

**Condition II.**

If the phase angles satisfy

$$|(\theta_0 - \theta_3) \mod 2\pi| = |(\theta_1 - \theta_2) \mod 2\pi| = \frac{\pi}{2} \tag{S6}$$

the UE-parameters and matrices become

$$a_0 = |a_0|\, e^{i\theta_0} = e^{i\theta_0}\sqrt{|f_0|^2 + |f_3|^2} \quad \text{and} \quad a_1 = |a_1|\, e^{i\theta_1} = e^{i\theta_1}\sqrt{|f_1|^2 + |f_2|^2}; \tag{S7}$$

$$V_0 = \begin{bmatrix} e^{i\zeta_0} & 0 \\ 0 & e^{-i\zeta_0} \end{bmatrix} \qquad \text{and} \qquad V_1 = \begin{bmatrix} 0 & e^{i\zeta_1} \\ e^{-i\zeta_1} & 0 \end{bmatrix}, \tag{S8}$$

where $\zeta_0$ and $\zeta_1$ are decided by

$$\cos\zeta_0 = \frac{|f_0|}{|a_0|} \quad \text{and} \quad \sin\zeta_0 = \frac{|f_3|}{|a_0|} e^{i(\theta_3-\theta_0-\frac{\pi}{2})} \tag{S9}$$

and

$$\cos\zeta_1 = \frac{|f_1|}{|a_1|} \text{ and } \sin\zeta_1 = \frac{|f_2|}{|a_1|} e^{i(\theta_2-\theta_1-\pi/2)}, \tag{S10}$$

respectively. From Eq. (S6), $e^{i(\theta_3-\theta_0-\pi/2)}$ and $e^{i(\theta_2-\theta_1-\pi/2)}$ in Eq. (S9) and (S10) are signs.

**Condition III.** If the phase angles satisfy

$$(\theta_2 - \theta_0) \mod \pi = (\theta_3 - \theta_1) \mod \pi = 0, \tag{S11}$$

the two UE-parameters and terms become

$$a_0 = |a_0|\, e^{i\theta_0} = e^{i\theta_0}\sqrt{|f_0|^2 + |f_2|^2} \quad \text{and} \quad a_1 = |a_1|\, e^{i\theta_3} = e^{i\theta_3}\sqrt{|f_1|^2 + |f_3|^2}; \tag{S12}$$

$$V_0 = \begin{bmatrix} \cos\zeta_0 & \sin\zeta_0 \\ -\sin\zeta_0 & \cos\zeta_0 \end{bmatrix} \quad \text{and} \quad V_1 = \begin{bmatrix} \cos\zeta_1 & \sin\zeta_1 \\ \sin\zeta_1 & -\cos\zeta_1 \end{bmatrix}, \tag{S13}$$

where $\zeta_0$ and $\zeta_1$ are decided by

$$\cos\zeta_0 = \frac{|f_0|}{|a_0|} \quad \text{and} \quad \sin\zeta_0 = \frac{|f_2|}{|a_0|} e^{i(\theta_2-\theta_0)} \tag{S14}$$

and

$$\cos\zeta_1 = \frac{|f_3|}{|a_1|} \quad \text{and} \quad \sin\zeta_1 = \frac{|f_1|}{|a_1|} e^{i(\theta_1-\theta_3)}, \tag{S15}$$

respectively. Notice that $e^{i(\theta_2-\theta_0)}$ and $e^{i(\theta_1-\theta_3)}$ are a signs.

**Condition IV.** If the phase angles satisfy

$$|(\theta_1-\theta_0) \mod 2\pi| = |(\theta_3-\theta_2) \mod 2\pi| = \frac{\pi}{2}, \tag{S16}$$

the UE-parameters and terms become

$$a_0 = |a_0|\, e^{i\theta_0} = e^{i\theta_0}\sqrt{|f_0|^2 + |f_1|^2} \quad \text{and} \quad a_1 = |a_1|\, e^{i\theta_3} = e^{i\theta_3}\sqrt{|f_2|^2 + |f_3|^2}; \tag{S17}$$

$$V_0 = \begin{bmatrix} \cos\zeta_0 & i\sin\zeta_0 \\ i\sin\zeta_0 & \cos\zeta_0 \end{bmatrix} \quad \text{and} \quad V_1 = \begin{bmatrix} \cos\zeta_1 & i\sin\zeta_1 \\ -i\sin\zeta_1 & -\cos\zeta_1 \end{bmatrix}, \tag{S18}$$

where $\zeta_0$ and $\zeta_1$ are decided by

$$\cos\zeta_0 = \frac{|f_0|}{|a_0|} \quad \text{and} \quad \sin\zeta_0 = \frac{|f_1|}{|a_0|} e^{i(\theta_1-\theta_0-\pi/2)} \tag{S19}$$

and

$$\cos\zeta_1 = \frac{|f_3|}{|a_1|} \quad \text{and} \quad \sin\zeta_1 = \frac{|f_2|}{|a_1|} e^{i(\theta_2-\theta_3-\pi/2)}, \tag{S20}$$

respectively. Noticing that $e^{i(\theta_1-\theta_0-\pi/2)}$ and $e^{i(\theta_2-\theta_3-\pi/2)}$ are equal to $\pm 1$.

**Special cases.** If one UE-parameter $f_k(t)$ in the main text is equal to zero, $F(t)$ can be expressed by two UE-terms in some special cases.

(a) In the case of $f_0(t) = 0$,

$$F(t) = e^{i\theta_3}\, |c_1|\, U_1 + e^{i\theta_3}\, |c_2|\, U_2. \tag{S21}$$

In fact, it is accordance with the *phase matching* condition I [see Eq. (S2)] if we set $f_0(t) = 0 \cdot e^{i\theta_3}$. From Eq. (S3) to (S5), it is not difficult to calculate the parameters and matrices in the main text that $a_0$, $a_1$, $V_0$ and $V_1$ are equal to $c_2$, $c_1$, $U_2$ and $U_1$, respectively.

(b) In the case of $f_1(t) = 0$, if $|(\theta_0-\theta_3) \mod 2\pi| = \pi/2$ or $(\theta_2-\theta_0) \mod \pi = 0$, either *phase matching* condition II [Eq. (S6)] or III [Eq. (S11)] is met, respectively.

(c) In the case of $f_2(t) = 0$, if $|(\theta_0-\theta_3) \mod 2\pi| = \pi/2$ or $|(\theta_1-\theta_0) \mod 2\pi| = \pi/2$, either *phase matching* condition II [Eq. (S6)] or IV [Eq. (S16)] is met, respectively.

(d) In the case of $f_3(t) = 0$, it naturally satisfies *phase matching* condition I [see Eq. (S2)] because $\theta_3$ can be set as $\theta_0$ and $f_3(t) = 0 \cdot e^{i\theta_0}$.

## Explicit expressions of UEs in the section of 'Illustration'

## PT-symmetric Hamiltonians.

A time-independent PT-symmetric two-level Hamiltonian has a general form of

$$H_{PT} = \begin{bmatrix} re^{i\theta} & se^{i\varphi} \\ se^{-i\varphi} & re^{-i\theta} \end{bmatrix}, \tag{S22}$$

satisfying $[PT, H_{PT}] = 0$, where $P = \sigma_1$ is the parity operator and $T$ is the time-reversal operator having the effect that $i \to -i$. The eigenvalue of $H_{PT}$ are $\varepsilon_\pm = rcos\theta \pm \sqrt{s^2 - r^2 \sin^2\theta}$, and we set the difference of them as

$$\omega = 2\sqrt{s^2 - r^2 \sin^2\theta}. \tag{S23}$$

$\omega$ is either real or imaginary depending on the system is in exact-PT or PT-broken phase, respectively. We now expand the time-evolution operator $e^{-i(t/\hbar)H_{PT}}$ as the form of Eq. (2) in the main text. The four UE-parameters are

$$f_0(t) = \left[\frac{1}{2}\left(e^{i\frac{\omega t}{2\hbar}} + e^{-i\frac{\omega t}{2\hbar}}\right)\right] e^{-i\frac{t}{\hbar} r\cos\theta}, \tag{S24}$$

$$f_1(t) = i\left[-\frac{s\cos\varphi}{i\omega}\left(e^{i\frac{\omega t}{2\hbar}} - e^{-i\frac{\omega t}{2\hbar}}\right)\right] e^{-i\frac{t}{\hbar} r\cos\theta}, \tag{S25}$$

$$f_2(t) = \left[\frac{s\sin\varphi}{i\omega}\left(e^{i\frac{\omega t}{2\hbar}} - e^{-i\frac{\omega t}{2\hbar}}\right)\right] e^{-i\frac{t}{\hbar} r\cos\theta}, \tag{S26}$$

and

$$f_3(t) = \left[\frac{r\sin\theta}{i\omega}\left(e^{i\frac{\omega t}{2\hbar}} - e^{-i\frac{\omega t}{2\hbar}}\right)\right] e^{-i\frac{t}{\hbar} r\cos\theta}. \tag{S27}$$

Terms in the brackets are always real, while terms outside the brackets are the phases. the phase angles $\theta_0$ and $\theta_3$ of $f_0$ and $f_3$ satisfy *phase matching* condition I in Eq. (S2).

## Anti-PT-symmetric Hamiltonians.

A Hamiltonian is anti-PT-symmetric if $\{PT, H_{APT}\} = 0$. In fact, the general form of the Hamiltonian is equal to the imaginary unit $i$ times $H_{PT}$. For a time-independent two-state system,

$$H_{APT} = i\begin{bmatrix} re^{i\theta} & se^{i\varphi} \\ se^{-i\varphi} & re^{-i\theta} \end{bmatrix} = iH_{PT}. \tag{S28}$$

The eigenvalues are $\varepsilon_\pm = i\left(rcos\theta \pm \sqrt{s^2 - r^2\sin^2\theta}\right)$, and we set the difference of them as

$$\lambda = 2\sqrt{r^2\sin^2\theta - s^2} = 2i\sqrt{s^2 - r^2\sin^2\theta} = i\omega, \tag{S29}$$

where $\omega$ is that in Eq. (S23). The unitary expansion of the time-evolution operator $e^{-i(t/\hbar)H_{APT}}$ can be expanded as Eq. (2) in the main text, and the four UE-parameters are

$$f_0(t) = \left[\frac{1}{2}\left(e^{i\frac{\lambda t}{2\hbar}} + e^{-i\frac{\lambda t}{2\hbar}}\right) e^{\frac{t}{\hbar} r\cos\theta}\right], \tag{S30}$$

$$f_1(t) = \left[\frac{s\cos\varphi}{i\lambda}\left(e^{i\frac{\lambda t}{2\hbar}} - e^{-i\frac{\lambda t}{2\hbar}}\right) e^{\frac{t}{\hbar} r\cos\theta}\right], \tag{S31}$$

$$f_2(t) = i\left[\frac{s\sin\varphi}{i\lambda}\left(e^{i\frac{\lambda t}{2\hbar}} - e^{-i\frac{\lambda t}{2\hbar}}\right) e^{\frac{t}{\hbar} r\cos\theta}\right], \tag{S32}$$

and

$$f_3(t) = i\left[\frac{r\sin\theta}{i\lambda}\left(e^{i\frac{\lambda t}{2\hbar}} - e^{-i\frac{\lambda t}{2\hbar}}\right) e^{\frac{t}{\hbar} r\cos\theta}\right]. \tag{S33}$$

Phases of the UE-parameters are outside the brackets, and they meets the *phase matching* condition II in Eq. (S6). Therefore, a time-independent anti-$PT$-symmetric two-level system can be simulated by two qubits.

## ***P*-pseudo-Hermitian Hamiltonians.**

A $P$-pseudo-Hermitian Hamiltonian, say $H_{PPH}$, satisfies $PH_{PPH}^{\dagger}P = H_{PPH}$. For a time-independent two-level system,

$$H_{PPH} = \begin{bmatrix} re^{i\theta} & s \\ u & re^{-i\theta} \end{bmatrix}, \tag{S34}$$

of which the eigenvalues are $\varepsilon_{\pm} = rcos\theta \pm \sqrt{us - r^2 \sin^2\theta}$. The difference of the two eigenvalues is

$$\omega = 2\sqrt{us - r^2 \sin^2\theta}. \tag{S35}$$

The time-evolution operator $e^{-i(t/\hbar)H_{PPH}}$ can be expanded as the form of Eq. (2) in the main text with the four UE-parameters as follow

$$f_0(t) = \left[\frac{1}{2}\left(e^{i\frac{\omega t}{2\hbar}} + e^{-i\frac{\omega t}{2\hbar}}\right)e^{\frac{t}{\hbar}r\cos\theta}\right]e^{-i\frac{t}{\hbar}r\cos\theta}, \tag{S36}$$

$$f_1(t) = i\left[-\frac{s+u}{2i\omega}\left(e^{i\frac{\omega t}{2\hbar}} - e^{-i\frac{\omega t}{2\hbar}}\right)e^{\frac{t}{\hbar}r\cos\theta}\right]e^{-i\frac{t}{\hbar}r\cos\theta}, \tag{S37}$$

$$f_2(t) = i\left[-\frac{s-u}{2i\omega}\left(e^{i\frac{\omega t}{2\hbar}} - e^{-i\frac{\omega t}{2\hbar}}\right)e^{\frac{t}{\hbar}r\cos\theta}\right]e^{-i\frac{t}{\hbar}r\cos\theta}, \tag{S38}$$

and

$$f_3(t) = \left[\frac{r\sin\theta}{i\omega}\left(e^{i\frac{\omega t}{2\hbar}} - e^{-i\frac{\omega t}{2\hbar}}\right)e^{\frac{t}{\hbar}r\cos\theta}\right]e^{-i\frac{t}{\hbar}r\cos\theta}. \tag{S39}$$

Comparing with *phase matching* conditions (see Sec. 2), the phases of the four UE-parameters meet both the condition I and IV in Eq. (S2) and (S16).

## **Anti-*P*-pseudo-Hermitian Hamiltonians**

Timed by the imaginary unit $i$, $H_{PPH}$ becomes an anti-$P$-pseudo-Hermitian (anti-PPH) Hamiltonian, which satisfies $PH_{APPH}^{\dagger}P = -H_{APPH}$. A time-independent two-dimensional one is

$$H_{APPH} = i\begin{bmatrix} re^{i\theta} & s \\ u & re^{-i\theta} \end{bmatrix} = iH_{PPH}. \tag{S40}$$

The eigenvalues are $\varepsilon_{\pm} = i\left(rcos\theta \pm \sqrt{us - r^2\sin^2\theta}\right)$, and the difference of them is

$$\lambda = 2\sqrt{r^2\sin^2\theta - us} = 2i\sqrt{us - r^2\sin^2\theta} = i\omega, \tag{S41}$$

where $\omega$ is that in Eq. (S35).

(i) In an arbitrary phase, the time-evolution operator $e^{-i(t/\hbar)H_{APPH}}$ has the form of Eq. (2) in the main text, where the four UE-parameters are equal to

$$f_0(t) = \left[\frac{1}{2}\left(e^{i\frac{\lambda t}{2\hbar}} + e^{-i\frac{\lambda t}{2\hbar}}\right)e^{\frac{t}{\hbar}r\cos\theta}\right], \tag{S42}$$

$$f_1(t) = \left[\frac{s+u}{2i\lambda}\left(e^{i\frac{\lambda t}{2\hbar}} - e^{-i\frac{\lambda t}{2\hbar}}\right)e^{\frac{t}{\hbar}r\cos\theta}\right], \tag{S43}$$

$$f_2(t) = \left[\frac{s-u}{2i\lambda}\left(e^{i\frac{\lambda t}{2\hbar}} - e^{-i\frac{\lambda t}{2\hbar}}\right)e^{\frac{t}{\hbar}r\cos\theta}\right], \tag{S44}$$

and

$$f_3(t) = i\left[\frac{r\sin\theta}{i\lambda}\left(e^{i\frac{\lambda t}{2\hbar}} - e^{-i\frac{\lambda t}{2\hbar}}\right)e^{\frac{t}{\hbar}r\cos\theta}\right]. \tag{S45}$$

Noticing that $\lambda$ is either real or imagine, the terms in the brackets are real. The phases of the four UE-parameters are 0, 0, 0 and $\pi/2$, so none of the *phase matching* conditions are met. Therefore, three qubits are necessary to simulate this system in a general case by either the six- or eight-dimension protocols.

(ii) In some special cases (see Sec. 2.5), the system can be simulated by two qubits. For example, when the system is in the anti-PT-symmetric phase, i.e. $s = u$, one of the UE-parameters $f_2(t)$ of the time-evolution operator $e^{-i(t/\hbar)H_{APPH}}$ becomes zero. As discussed in the first section above, it meets both the *phase matching* condition II [Eq. (S6)] and IV [Eq. (S16)]. So the system can be simulated by two qubits in this anti-PT-symmetric phase.

## Single-qubit measurements: $\sigma_3$-measurement.

As a simple case, we illustrate how to simulate a single-qubit $\sigma_3$-measurement. Assuming $|0\rangle$ and $|1\rangle$ are the two eigen states of $\sigma_3$, the effect of this measurement is to apply either of the two nonunitary matrices

$$M_0 = |0\rangle\langle 0| = \begin{bmatrix} 1 & 0 \\ 0 & 0 \end{bmatrix} = \frac{1}{2}(\sigma_0 + \sigma_3) \qquad \text{or} \qquad M_1 = |1\rangle\langle 1| = \begin{bmatrix} 0 & 0 \\ 0 & 1 \end{bmatrix} = \frac{1}{2}(\sigma_0 - \sigma_3) \tag{S46}$$

with probabilities ${}_e\langle 0|\psi\rangle_e$ or ${}_e\langle 1|\psi\rangle_e$, respectively. We now apply a $U = H_2$, $C_{0-\sigma_0}$, $C_{1-\sigma_3}$ and another $H_2$ as quantum circuit in Fig.(6) in the main text. Now, the system evolves to a state

$$|0\rangle_a M_0|\psi\rangle_e + |1\rangle_a M_1|\psi\rangle_e. \tag{S47}$$

Finally, a measurement is performed on the ancillary qubit. If an output $|k\rangle_a$ is obtained, the work qubit will evolve to $|k\rangle_e$ with a probability of ${}_e\langle k|\psi\rangle_e$ $(k = 0, 1)$. Although the ancillary qubit is annihilated, the work qubit is kept for further use.

## The Abrams–Lloyd's gate

The explicit forms of the UE-terms and parameters of $N = \sum_{k=0}^{4} n_k N_k$ are unitary matrices

$$N_0 = \begin{bmatrix} \frac{1}{\sqrt{2}\sqrt{2-\sqrt{2}}}\sigma_0 & -\frac{\sqrt{2}-1}{\sqrt{2}\sqrt{2-\sqrt{2}}}\sigma_0 \\ \frac{\sqrt{2}-1}{\sqrt{2}\sqrt{2-\sqrt{2}}}\sigma_0 & \frac{1}{\sqrt{2}\sqrt{2-\sqrt{2}}}\sigma_0 \end{bmatrix}, \tag{S48}$$

$$N_1 = \begin{bmatrix} -\frac{1}{2}(i\sigma_2) & \frac{1}{2}\sigma_0 \\ -\frac{1}{2}(i\sigma_2) & -\frac{1}{2}\sigma_0 \end{bmatrix}, \tag{S49}$$

$$N_2 = \begin{bmatrix} -\frac{1}{\sqrt{2}}(\sigma_3 + \sigma_1) & 0 \\ 0 & \sigma_0 \end{bmatrix}, \tag{S50}$$

$$N_3 = \begin{bmatrix} 0 & \sigma_0 \\ -\frac{1}{\sqrt{2}}(\sigma_3 + \sigma_1) & 0 \end{bmatrix} \tag{S51}$$

and

$$N_4 = \begin{bmatrix} -\frac{1}{\sqrt{2}}\sigma_0 & \frac{1}{\sqrt{2}}\sigma_0 \\ \frac{1}{\sqrt{2}}\sigma_0 & \frac{1}{\sqrt{2}}\sigma_0 \end{bmatrix}; \tag{S52}$$

$n_0 = \sqrt{2-\sqrt{2}}$, $n_1 = 1$, $n_2 = n_3 = 1/\sqrt{2}$, $n_4 = (\sqrt{2}-1)/\sqrt{2}$ are the UE-parameters.

The normalizing factor in the main text is

$$f_N = \sqrt{\sum_{k=0}^{4} |n_k|^2} = \sqrt{\frac{11}{2} - 2\sqrt{2}} \tag{S53}$$
